# A new essential protein discovery method based on the integration of protein-protein interaction and gene expression data

**DOI:** 10.1186/1752-0509-6-15

**Published:** 2012-03-10

**Authors:** Min Li, Hanhui Zhang, Jian-xin Wang, Yi Pan

**Affiliations:** 1 School of Information Science and Engineering, Central South University, Changsha, Hunan 410083, P. R. China; 2 Department of Computer Science, Georgia State University, Atlanta, GA 30302-4110, USA

## Abstract

**Background:**

Identification of essential proteins is always a challenging task since it requires experimental approaches that are time-consuming and laborious. With the advances in high throughput technologies, a large number of protein-protein interactions are available, which have produced unprecedented opportunities for detecting proteins' essentialities from the network level. There have been a series of computational approaches proposed for predicting essential proteins based on network topologies. However, the network topology-based centrality measures are very sensitive to the robustness of network. Therefore, a new robust essential protein discovery method would be of great value.

**Results:**

In this paper, we propose a new centrality measure, named PeC, based on the integration of protein-protein interaction and gene expression data. The performance of PeC is validated based on the protein-protein interaction network of *Saccharomyces cerevisiae*. The experimental results show that the predicted precision of PeC clearly exceeds that of the other fifteen previously proposed centrality measures: Degree Centrality (DC), Betweenness Centrality (BC), Closeness Centrality (CC), Subgraph Centrality (SC), Eigenvector Centrality (EC), Information Centrality (IC), Bottle Neck (BN), Density of Maximum Neighborhood Component (DMNC), Local Average Connectivity-based method (LAC), Sum of ECC (SoECC), Range-Limited Centrality (RL), L-index (LI), Leader Rank (LR), Normalized *α*-Centrality (NC), and Moduland-Centrality (MC). Especially, the improvement of PeC over the classic centrality measures (BC, CC, SC, EC, and BN) is more than 50% when predicting no more than 500 proteins.

**Conclusions:**

We demonstrate that the integration of protein-protein interaction network and gene expression data can help improve the precision of predicting essential proteins. The new centrality measure, PeC, is an effective essential protein discovery method.

## Background

The identification of essential proteins is crucial for understanding of the minimal requirements for cellular life [1], which is also very important for the discovery of human disease genes and defending against human pathogens [2-4]. For example, the identification of essential genes and non-essential genes is valuable for rational drug design [5]. Essential proteins in pathogenic organisms can be taken as the potential targets for new antibiotics [6].

Essential proteins are those proteins necessary for growth in a rich medium where all the required nutrients are available [1]. The deletion of such proteins will result in lethality or infertility, i.e., the organism cannot survive without them [7,8]. Different experimental methods, such as single gene knockouts [9], RNA interference [10] and conditional knockouts [11], have been implemented for the discovery of essential proteins. However, these experimental methods generally require large amounts of resources and are very time consuming.

To break through these experimental constraints, some researchers have proposed various computational approaches. With the accumulation of data derived from experimental small-scale studies and high-throughput techniques, there is a growing awareness that the topological properties of biological networks would be useful for the identification of essential proteins. It has been observed in several species, such as *Saccharomyces cerevisiae, Caenorhabditis elegans*, and *Drosophila melanogaster *[12,13], that proteins in the network highly connecting with other proteins are more likely to be essential than those selected by chance [14]. This is called the "centrality-lethality rule" [14]. Although there exist some controversies about whether, why and how the highly connected proteins tend to be essential in biological networks [15-18], most researchers have confirmed the correlation between topological centrality and protein essentiality [13,19-21].

Specifically, some global network characteristics, such as betweenness centrality [22] and closeness centrality [23], and local network features, such as maximum neighborhood component [24] and local average connectivity [25], have already been used to determine a protein's essentiality. Recently, Park and Kim [26] investigated the localized network centrality and essentiality in the yeast protein-protein interaction network. They made a comprehensive examination and comparison among different types of centrality measures, which included shortest path betweenness, shortest path closeness, eigenvector centrality, harary graph centrality, information centrality, stress centrality, random walk betweenness, random walk closeness, degree centrality, clustering coefficient, subgraph centrality, complexity measure, sub-network maximum degree, and assortative mixing (ASS) centralities. In our previous studies [25,27,28], we have also shown the feasibility of using network topological features to detect essential proteins from the yeast protein-protein interaction networks. Moreover, several recent centrality measures, such as Range-Limited Centrality [29], L-Index [30], LeaderRank [31], Normalized *α*-Centrality [32], and Moduland-Centrality [33], used in complex networks can also be used to analyze the protein-protein interaction networks.

Though a great progress has been made on the computational methods for the identification of essential proteins based on network topologies, there are still several challenges that researchers have to meet. First, the protein-protein interaction dataset for each species is not complete up to now. Second, a high proportion of false positives has been found in protein-protein interaction networks, especially for those obtained by high-throughput technologies. In addition, as reported by Zotenko et al. [17], essential proteins tend to form highly connected clusters rather than function independently. It is well known that both false negatives and false positives in protein-protein interaction networks are hard to be cleaned out. For false positives, a general approach is to evaluate the interactions by using different weighting methods. More recently, there is a new trend that improves the precision of essential protein discovery method by integration of network topology and other information. For example, Acencio et al [1] explored essential proteins based on the integration of network topological features and two types of GO annotations: cellular localization and biological process. Recently, several researchers began to pay attention to the relationship between protein essentiality and their cluster property [27,34].

With respect to these various difficulties and progresses, we propose a new centrality measure, named PeC, by integrating protein-protein interaction data and gene expression data. Different from other centrality measures, PeC determines a protein's essentiality not only based on its connectivity, but also whether it has a high probability to be co-clustered and co-expressed with its neighbors. The performance of PeC was tested on the well studied species of *Saccharomyces cerevisiae*. Compared to other fifteen previous centrality measures: Degree Centrality (DC) [14], Betweenness Centrality (BC) [22], Closeness Centrality (CC) [23], Subgraph Centrality(SC) [35], Eigenvector Centrality(EC) [36], Information Centrality(IC) [37], Bottle Neck (BN) [38,39], Density of Maximum Neighborhood Component (DMNC) [24], Local Average Connectivity-based method (LAC) [25], Sum of ECC (SoECC) [27], Range-Limited Centrality (RL) [29], L-Index (LI) [30], LeaderRank (LR) [31], Normalized *α*-Centrality (NC) [32], and Moduland-Centrality (MC) [33], PeC achieves higher precision for the identification of essential proteins. The experimental results show that the integration of network topology and gene expression increased the predictability of essential proteins in comparison with those centrality measures only based on network topological features.

### New centrality measure: PeC

In this study, a new centrality measure, PeC, is proposed based on the integration of protein-protein interaction data and gene expression data. The basic ideas behind PeC are as follows: (1) A highly connected protein is more likely to be essential than a low connected one; (2) Essential proteins tend to form densely connected clusters; (3) Essential proteins in the same cluster have a more chance to be co-expressed. In PeC, a protein's essentiality is determined by the number of the protein's neighbors and the probability that the protein is co-clustered and co-expressed with its neighbors.

To describe PeC simply and clearly, we provide the following definitions and descriptions. The protein-protein interaction network is represented by an undirected graph *G*(*V, E*), where a node *v *∈ *V *represents a protein and an edge *e*(*u, v*) ∈ *E *denotes an interaction between two proteins *u *and *v*. Gene expression is the process by which information from a gene is used in the synthesis of a functional gene product. These gene products are often proteins. Of course, there may exist some functional RNAs from non-protein coding genes. Here, we only consider the gene expressions for proteins. For a protein *v*, its gene expressions with *s *different times are denoted as *Ge(v) *= {*g*(*v*, 1), *g*(*v*, 2), ..., *g*(*v, s*)}. The probability that two proteins are co-clustered and co-expressed is evaluated based on the edge clustering coefficient (ECC) and pearson correlation coefficient (PCC).

### Edge clustering coefficient *(ECC)*

Clustering coefficient was first proposed to describe the property of a vertex in a network, which has been used as an effective tool to analyze the topology of protein-protein interaction networks [40]. Radicchi *et al*. [41] generalized the clustering coefficient of a vertex to an edge, and defined it as the number of triangles to which a given edge belonged, divided by the number of triangles that might potentially include the triangles. In our previous studies [25,42], we have proposed a modified definition of edge clustering coefficient (ECC) to overcome the fact that the definition of ECC in [41] is not feasible when the network has few triangles. For an edge (*u, v*) connecting node *u *and node *v*, we calculate its ECC by using the common neighbors instead of triangles. The ECC of an edge (*u, v*) is defined as:

(1) $$ECC\left(u,\;v\right)=\frac{|N_{u}\cap N_{v}|+1}{min\left\{d_{u},d_{v}\right\}}$$

where *N_u _*(or *N_v_*) is the set of neighbors of vertex *u *(or *v*) and *d_u _*(or *d_v_*) denotes the degree of vertex *u *(or *v*), i.e., the number of nodes which *u *(or *v*) directly connects in graph *G*.

*ECC*(*u, v*) is a local variable which characterizes the closeness of two proteins *u *and *v*. Obviously, two proteins *u *and *v *with a larger value of *ECC*(*u, v*) are more likely to be in the same cluster.

The advantage of *ECC *is that it describes effectively the probability of two proteins being in a cluster from the topology view. However, it also has disadvantage. The effectiveness of *ECC *heavily depends on the reliability of the protein-protein interaction networks. Thus, in this paper we will introduce another metric, pearson correlation coefficient, which is independent of the reliability of the protein-protein interaction networks, to evaluate how likely two proteins are in the same cluster from another view.

### Pearson correlation coefficient (PCC)

To evaluate how strong two interacting proteins are co-expressed, we calculate their pearson's correlation coefficient(PCC). The PCC [43] of a pair of genes (*X *and *Y*), which encode the corresponding paired proteins (*u *and *v*) interacting in the protein-protein interaction network, is defined as:

(2) $$PCC\left(X,Y\right)=\frac{1}{s-1}\sum_{t=1}^{s}\;\;\left(\frac{g\left(X,i\right)-ḡ\left(X\right)}{\sigma \left(X\right)}\right).\left(\frac{g\left(Y,i\right)-ḡ\left(Y\right)}{\sigma \left(Y\right)}\;\right)$$

where *s *is the number of samples of the gene expression data; *g*(*X, i*) (or (*g*(*Y, i*))) is the expression level of gene *X *(or *Y*) in the sample *i *under a specific condition; *ḡ*(*X*) (or *ḡ *(*Y*)) represents the mean expression level of gene *X *(or *Y*) and *σ*(*X*) (or *σ*(*Y*)) represents the standard deviation of expression level of gene *X *(or *Y)*. Here, we defined the pearson's correlation coefficient of a pair of proteins (*u *and *v*) as equal to the PCC of their corresponding paired genes (*X *and *Y*), that is *PCC*(*u, v*) = *PCC*(*X, Y*). The value of *PCC *ranges from -1 to 1. If *PCC*(*u, v*) has a positive value, there is a positive linear correlation between *u *and *v*.

### New centrality measure PeC by integration of PCC and ECC

It has been proved that there exist a number of protein complexes which play a key role in carrying out biological functionality [44] and the essentiality tends to be a product of a protein complex rather than an individual protein [45]. Based on the definitions of edge clustering coefficient (ECC) and pearson's correlation coefficient (PCC), we propose a new centrality measure which is named as PeC. The probability that two proteins are co-clustered is described from a topological view and the probability that two proteins are co-clustered is characterized from a biological view. Thus, we defined the probability of paired proteins *u *and *v *to be in the same cluster as following:

(3) $$p_{c}\left(u,v\right)=ECC\left(u,v\right)\times PCC\left(u,v\right)$$

For a protein *v*, its *PeC(v) *is defined as the sum of the probabilities that the protein and its neighbors belong to a same cluster:

(4) $$PeC\left(v\right)=\sum_{u\in N_{v}}\;\;p_{c}\left(u,v\right)$$

Where *N_v _*denotes the set of all neighbors of node *v*.

The value of *PeC(v) *is determined by not only how many neighbors the protein has but also how likely it is co-clustered with its neighbors. In our previous studies [25], we have found that in the cases of non-essential proteins, which have a high degree, there are generally few interactions between their neighbors. When predicting essential proteins, PeC can discriminate these different types of highly connected proteins by the computation of sum of *p_c_*.

## Results and discussion

### Test data

To evaluate the performance of the proposed new centrality measure, PeC, we implemented it on the discovery of essential proteins of *Saccharomyces cerevisiae*, as it has been well characterized by knockout experiments and widely used in the evaluations of essential proteins. The test data used in this paper are as following:

#### Protein-protein interaction data

The protein-protein interactions of *Saccharomyces cerevisiae *was downloaded from the DIP database [46]. There are 24,743 interactions among 5093 proteins in total after the self-interactions and the repeated interactions were filtered.

#### Essential proteins

A list of essential proteins of *Saccharomyces cerevisiae *were collected from the following databases: MIPS [47], SGD [48], DEG [49], and SGDP [50]. A protein in the yeast protein interaction network is considered as an essential protein if it is marked as essential at least in one database. Out of all the 5093 proteins in the yeast network, 1167 proteins are essential, 3591 are non-essential, and the rest 335 are still unknown to be essential or non-essential.

#### Gene expression

The gene expression data of *Saccharomyces cerevisiae *was retrieved from Tu et al., 2005 [51], containing 6,777 gene products and 36 samples in total, with 4,858 genes involved in the yeast protein interaction network.

The detailed information of proteins with gene expression data is shown in Additional file 1.

### Compare PeC with other centrality measures

To validate the performance of the proposed new centrality measure PeC, we carry out a comparison between it and fifteen other previously proposed centrality measures: Degree Centrality (DC) [14], Betweenness Centrality (BC) [22], Closeness Centrality (CC) [23], Subgraph Centrality(SC) [35], Eigenvector Centrality(EC) [36], Information Centrality(IC) [37], Bottle Neck (BN) [38,39], Density of Maximum Neighborhood Component (DMNC) [24], Local Average Connectivity-based method (LAC) [25], Sum of ECC (SoECC) [27], Range-Limited Centrality (RL) [29], L-Index (LI) [30], LeaderRank (LR) [31], Normalized *α*-Centrality (NC) [32], and Moduland-Centrality (MC) [33].

**Figure 1:**
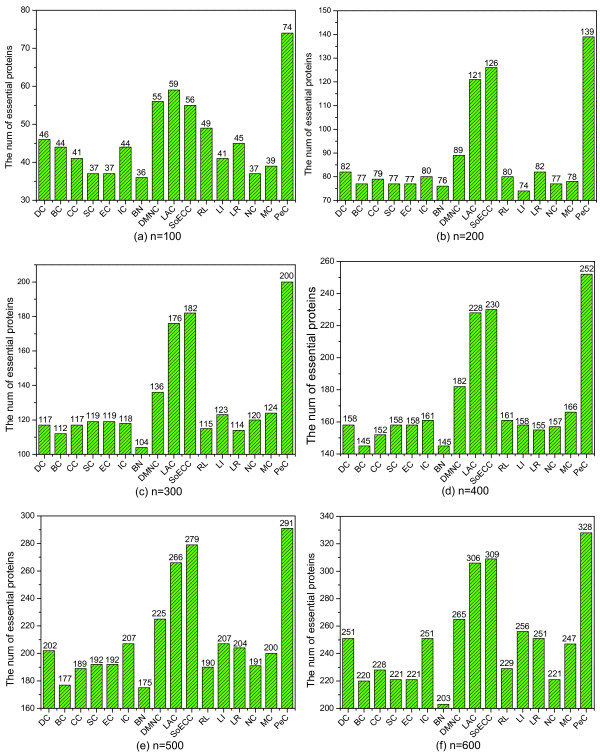
**Comparison of the number of essential proteins detected by PeC and fifteen other previously proposed centrality measures**. For each centrality measure, a certain number of top proteins are selected as candidates for essential proteins and out of which the number of true essential proteins are determined. The number of true essential proteins detected by PeC and fifteen other previously proposed centrality measures: Degree Centrality(DC), Betweenness Centrality (BC), Closeness Centrality (CC), Subgraph Centrality(SC), Eigenvector Centrality(EC), Information Centrality(IC), Bottle Neck (BN), Density of Maximum Neighborhood Component (DMNC), Local Average Connectivity-based method (LAC), Sum of ECC (SoECC), Range-Limited Centrality(RL), L-index(LI), Leader Rank(LR), Normalized *α*-Centrality(NC), and Moduland-Centrality(MC) from the yeast protein-protein interaction network are shown.

Proteins are ranked according to their values calculated by each centrality measure. A certain number of top proteins are selected as candidates for essential proteins. Then we determine how many of them are true essential proteins. The number of essential proteins detected by PeC and fifteen other centrality measures (DC, BC, CC, SC, EC, IC, BN, DMNC, LAC, SoECC, RL, LI, LR, NC, and MC) from the yeast protein-protein interaction network is shown in Figure 1.

From Figure 1 we can see that PeC performs significantly better than all the fifteen previous aforementioned centrality measures for predicting essential proteins from the yeast protein interaction network. Especially, the improvement of PeC over the classic centrality measures (BC, CC, SC, EC, and BN) is more than 50%. Even so, there is about 10% improvement of PeC compared to LAC and SoECC.

### Validated by jackknife methodology

A more general comparison between the proposed new centrality measure PeC and the fifteen previously proposed centrality measures (DC, BC, CC, SC, EC, IC, BN, DMNC, LAC, SoECC, RL, LI, LR, NC, and MC) is tested by using a jackknife methodology [52]. The comparison results are shown in Additional file 2. There are five figures in the Additional file 2: (a) shows the comparison result of PeC and two local metric-based centrality measures: DC andDMNC; (b) shows the comparison result of PeC and three centrality measures: BC, SC and BN; (c) shows the comparison result of PeC and three classic centrality measures: IC, EC, and CC; (d) shows the comparison result of PeC and our previously proposed two methods: LAC and SoECC; (e) shows the comparison result of PeC and five recent centrality measures: RL, LI, LR, NC, and MC. In Additional file 2, proteins are ordered from the highest value to the lowest value for each centrality measure and the cumulative counts of essential proteins are plotted. The areas under the curve (AUC) for PeC and that for other previously proposed centrality measures are compared. In addition, ten random assortments are also plotted for comparison.

As shown in Additional file 2, it is clear that the sorted curve of PeC appears to be much better than that of the fifteen previously proposed centrality measures: DC, BC, CC, SC, EC, IC, BN, DMNC, LAC, SoECC, RL, LI, LR, NC, MC and all the results of these centrality measures are better than that of randomized sorting. The comparison results shown in Additional file 2 indicate that the integration of protein-protein interaction and gene expression data can help improve the predicted precision of identifying essential proteins.

### Analysis of the differences between PeC and other centrality measures

To further analyze why and how PeC performs well on the identification of essential proteins we study the relationship and difference between it and fifteen other centrality measures (DC, BC, CC, SC, EC, IC, BN, DMNC, LAC, SoECC, RL, LI, LR, NC, and MC) by predicting a small fraction of proteins. For each centrality measure, the top 100 proteins are selected. The information of the top 100 proteins of PeC and fifteen other centrality measures is shown in Additional file 3.

Firstly, we compare PeC with DC, BC, CC, SC, EC, IC, BN, DMNC, LAC, SoECC, RL, LI, LR, NC, and MC by investigating how many proteins are both predicted by PeC and by anyone of the fifteen centrality measures. The number of overlaps between PeC and one of the other centrality measures is shown in Table 1. In Table 1, |*PeC *∩ *M_i_*| denotes the number of common proteins detected by PeC and by a centrality measure *M_i_*, {*M_i _*- *PeC*} means the set of proteins identified by *M_i _*not by *PeC*, and |*M_i _*- *PeC*| is the number of proteins identified by *M_i _*not by *PeC*.

**Table 1:** The relationships between PeC and fifteen other centrality measures for predicting the top 100 proteins.

Centrality measures (*M*_i_)	*|PeC *∩ *M*_i_|	|*M_i _*- *PeC*|	Non-essential proteins in {*M_i _*- *PeC*}	Percentage of non-essential proteins in {*M_i _*- *PeC*} with low PeC
Degree Centrality (DC)	18	82	44	54.5%
Betweenness Centrality (BC)	16	84	47	51.1%
Closeness Centrality (CC)	16	84	51	56.9%
Subgraph Centrality(SC)	11	89	59	64.4%
Eigenvector Centrality(EC)	11	89	59	64.4%
Information Centrality(IC)	17	83	47	55.3%
Bottle Neck (BN)	16	84	53	45.3%
Density of Maximum Neighborhood Component (DMNC)	12	88	42	42.9%
Local Average Connectivity-based method (LAC)	34	66	37	59.5%
Sum of ECC (SoECC)	37	63	31	54.8%
Range-Limited Centrality (RL)	17	83	42	54.8%
L-index (LI)	13	87	55	58.2%
Leader Rank(LR)	16	84	46	52.2%
Normalized *α*-Centrality (NC)	11	89	59	64.4%
Moduland-Centrality(MC)	11	89	57	66.7%

The relationships between PeC and fifteen other centrality measures (DC, BC, CC, SC, EC, IC, BN, DMNC, LAC, SoECC, RL, LI, LR, NC, and MC) are studied by evaluating the overlaps between their predicted proteins. For each centrality measure, the top 100 proteins are selected. Then, the number of proteins both predicted by PeC and by anyone of the other centrality measures are calculated.

From Table 1, we can see that the common proteins identified by PeC and DC, BC, CC, SC, EC, IC, BN, DMNC, RL, LI, LR, NC, MC are all less than 20%, and that common proteins both predicted by PeC and LAC, SoECC are less 40%. Such a small overlap between the predicted proteins of PeC and DC, BC, CC, SC, EC, IC, BN, DMNC, RL, LI, LR, NC, MC shows that PeC is a special centrality measure which is much different from others.

**Figure 2:**
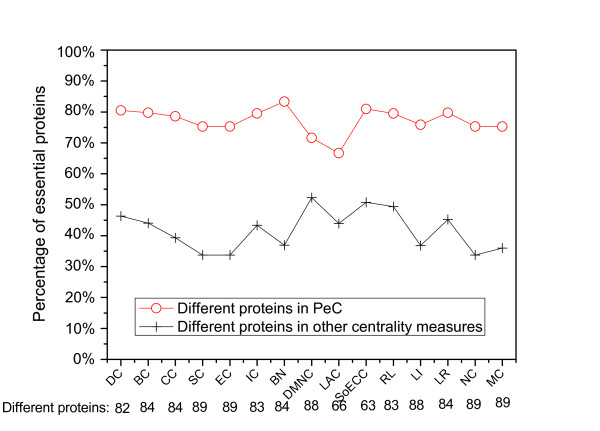
**Comparison of the percentage of essential proteins out of all the different proteins between PeC and fifteen other centrality measures: DC, BC, CC, SC, EC, IC, BN, DMNC, LAC, SoECC, RL, LI, LR, NC, and MC**. Figure 2 shows how many of the different proteins between PeC and fifteen other previously proposed centrality measures: Degree Centrality (DC), Betweenness Centrality (BC), Closeness Centrality (CC), Subgraph Centrality(SC), Eigenvector Centrality(EC), Information Centrality(IC), Bottle Neck (BN), Density of Maximum Neighborhood Component (DMNC), Local Average Connectivity-based method (LAC), Sum of ECC (SoECC), Range-Limited Centrality(RL), L-index(LI), Leader Rank(LR), Normalized *α*-Centrality(NC), and Moduland-Centrality(MC) are essential.

Secondly, we evaluate the different proteins identified by PeC and those by other centrality measures. Figure 2 shows how many essential proteins are predicted out of all the different proteins identify by PeC and those identified by DC, BC, CC, SC, EC, IC, BN, DMNC, LAC, SoECC, RL, LI, LR, NC, and MC. As expected, the results shown in Figure 2 illustrates that the percentage of essential proteins identified by PeC is consistently higher than that explored by fifteen other centrality measures for the different proteins between them. Take SC and SoECC as two extreme examples. The former has the largest different number of proteins from PeC, and the latter has the smallest difference from PeC. Out of all the top 100 proteins 89 are differently identified by SC and by PeC, respectively. Out of these 89 proteins of PeC, 75.3% ones are essential. In contrast, only 33.7% proteins identified by SC are essential. For another case, there are 63 different proteins identified by PeC and by SoECC. Out of 63 different proteins, PeC identified 80.9% essential proteins and SoECC only explored 50.8% essential proteins. The similar results are obtained from the rest centrality measures: DC, BC, CC, EC, IC, BN, DMNC, LAC, RL, LI, LR, NC, and MC.

A list of proteins which are predicted by PeC but ignored by all the ten centrality measures (DC, BC, CC, SC, EC, IC, BN, DMNC, LAC, and SoECC) when predicting the top 100 proteins are shown in Additional file 4. There are 41 proteins of PeC which are not included in any of the top 100 proteins of the ten other centrality measures. As shown in Additional file 4, out of the 41 proteins 83% are essential. In addition, we investigated the non-essential proteins predicted by other centrality measures and found that about 50% of them are with very low values of PeC (less than 0.55), as shown in Table 1.

Additional file 5 shows a list of non-essential proteins which have a high degree but with a low value of PeC. To further study the characteristics of these non-essential proteins, we also show in Additional file 5 their values of SoECC, SoPCC, average of ECC, and average of PCC. For a protein, its SoPCC is the sum of PCC between it and all its neighbors in the yeast protein-protein interaction network. The average of ECC and PCC describes how strongly a protein co-clustered or co-expressed withits neighbors, respectively. As shown in Additional file 5, all these non-essential proteins with a high degree consistently have a very low value of PeC. Take proteins YGR254W and YDL059C for examples. They both have a high degree of 67, as shown in Additional file 6, but both of them have few interactions in their neighbors and thus have a low average of ECC and PeC. Additional file 7 provides another two examples of non-essential proteins (YHR140W and YML048W) which not only have a high degree but also have a high value of SoECC. As shown in Additional file 7, YHR140W and YML048W are both included in a densely connected cluster. Though YHR140W and YML048W have a high probability to be co-clustered with their neighbors, they are not actually co-expressed with their neighbors. Besides YHR140W and YML048W, a list of other proteins which have a high degree and a high value of SoECC but with a low value of PeC is shown in Additional file 8. The results shown in Additional file 5, Additional file 6, Additional file 7, and Additional file 8 indicate that PeC can help filter the false predictions of other centrality measures.

## Conclusion

The identification of essential proteins from the network level is a hot topic in the postgenome era. Many approaches based on topological characteristics have been proposed for predicting essential proteins in biological networks. Unfortunately, most of the topology-based methods depend on the reliability of the available protein-protein interactions and thus are very sensitive to the network. To overcome these difficulties, we propose a new centrality measure, named PeC, based on the integration of protein-protein interaction and gene expression data. PeC is applied to the protein-protein interaction network of *Saccharomyces cerevisiae*. The experimental results show that the predicted precision of PeC is clearly higher than those of the fifteen other topology-based centrality measures: Degree Centrality (DC), Betweenness Centrality (BC), Closeness Centrality (CC), Subgraph Centrality(SC), Eigenvector Centrality(EC), Information Centrality(IC), Bottle Neck (BN), Density of Maximum Neighborhood Component (DMNC), Local Average Connectivity-based method (LAC), Sum of ECC (SoECC), Range-Limited Centrality(RL), L-index(LI), Leader Rank(LR), Normalized *α*-Centrality(NC), and Moduland-Centrality(MC).

Though PeC performs well on the identification of essential proteins, there may be still a space to improve the prediction performance. First, the integration of PCC and ECC is very simple in this paper. Further study on the relationship between PCC and ECC will provide new clues to integrating PCC and ECC in a more accurate way. Second, some other protein related data, such as biological process, domain information, and localization, besides the gene expression data, can also be integrated into the protein-protein interaction networks for identifying essential proteins. The integration of multiple protein related data may contribute a good deal to the identification of essential proteins with further research efforts.

## Competing interests

The authors declare that they have no competing interests.

## Authors' contributions

ML and HZ obtained the protein-protein interaction data, essential proteins and gene expression data. ML and HZ designed the new centrality, PeC, and analyzed the results. ML and HZ drafted the manuscript together. JW and YP participated in revising the draft. All authors have read and approved the manuscript.

## Supplementary Material

Additional file 1 **Information of the yeast protein-protein interaction network obtained from the DIP database**. This file shows the number of proteins, essential proteins, non-essential proteins, and interactions of the yeast protein-protein interaction network obtained from the DIP database. (DOC 28 kb). Click here for file

Additional file 2 **PeC is compared with fifteen recent centrality measures (DC, DMNC, BC, SC, BN, CC, EC, IC, LAC, SoECC, RL, LI, LR, NC, and MC) by a jackknife methodology**. This file includes five figures: (a) PeC is compared with DC and DMNC; (b) PeC is compared with BC, SC and BN; (c) PeC is compared with CC, EC and IC; (d) PeC is compared with LAC and SoECC; (e) PeC is compared with RL, LI, LR, NC, and MC. To compare with the results of random sorting, ten random assortments are also plotted in each figure. The X-axis represents the ranked proteins in the yeast protein-protein interaction network, ranked from left to right as the highest to the lowest values of centrality measures. The Y-axis is the cumulative count of essential proteins with respect to the ranked proteins moving left to right. (DOC 7744 kb). Click here for file

Additional file 3 **The top 100 proteins identified by PeC and other ten centrality measures**. This file is composed by 11 groups of data corresponding to PeC and other ten centrality measures: Degree Centrality (DC), Betweenness Centrality (BC), Closeness Centrality (CC), Subgraph Centrality(SC), Eigenvector Centrality(EC), Information Centrality(IC), Bottle Neck (BN), Density of Maximum Neighborhood Component (DMNC), Local Average Connectivity-based method (LAC), Sum of ECC (SoECC). (XLS 36 kb). Click here for file

Additional file 4 **A list of 41 proteins predicted by PeC which are ignored by the ten centrality measures: DC, DMNC, BC, SC, BN, CC, EC, IC, LAC, SoECC when predicting the top 100 proteins**. There are some proteins which are ignored by the ten centrality measures: DC, BC, CC, SC, EC, IC, BN, DMNC, LAC, and SoECC, but identified by PeC. This file provides the list of 41 proteins predicted by PeC which are ignored by all the ten centrality measures when predicting the top 100 proteins. (DOC 68 kb). Click here for file

Additional file 5 **A list of 25 non-essential proteins with a low value of PeC predicted by DC**. The non-essential proteins predicted by DC which have a low value of PeC are shown in this file. For each non-essential protein, its values of SoECC, SoPCC, average of ECC, and average of PCC are also shown in this file. (XLS 17 kb). Click here for file

Additional file 6 **Examples of non-essential proteins which have high degree but with low PeC**. Two examples of non-essential proteins YGR254W and YDL059C are shown. YGR254W and YDL059C both have a high degree of 67, but their PeC values are very low. The PeC value of YGR254W is 0.007 and that of YDL059C is -0.241. (DOC 246 kb). Click here for file

Additional file 7 **Examples of non-essential proteins which have high degree and high SoECC but with low PeC**. Two examples of non-essential proteins YML048W and YHR140W are shown. YML048W and YHR140W not only have a high degree but also have a high value of SoECC. However, their PeC values are very low. The PeC of YML048W is -0.241 and that of YHR140W is -2.447. (DOC 518 kb). Click here for file

Additional file 8 **A list of 17 non-essential proteins with a low value of PeC predicted by SoECC**. The non-essential proteins predicted by SoECC which have a low value of PeC are shown in this file. For each non-essential protein, its values of SoECC, SoPCC, average of ECC, and average of PCC are also shown in this file. (XLS 24 kb). Click here for file
