# Supplementary material for: A new essential protein discovery method based on the integration of protein-protein interaction and gene expression data
Source: BMC Syst Biol. 2012 Mar 10;6:15. doi: 10.1186/1752-0509-6-15 (PMC3325894; doi:10.1186/1752-0509-6-15)
Supplement: Additional file 1 — Information of the yeast protein-protein interaction network obtained from the DIP database. This file shows the number of proteins, essential proteins, non-essential proteins, and interactions of the yeast protein-protein interaction network obtained from the DIP database. (DOC 28 kb). [file 1752-0509-6-15-S1.DOC]

Table A - Information of the yeast protein-protein interaction network obtained from the DIP database.

| Number of proteins | 5093 |
| --- | --- |
| Number of interactions | 24743 |
| The average degree | 9.72 |
| Number of essential proteins | 1167 |
| Number of non-essential proteins | 3591 |
| Number of essentiality unknown proteins | 335 |
| Number of proteins with gene expression data | 4858 |
| Number of proteins without gene expression data | 235 |

For these proteins without any gene expression information, their gene expression levels are considered as zero by default. For a pair of proteins *u* and *v*, we only calculate their ECC if they both do not have any gene expression data on 36 samples.
