# Supplementary material for: A new essential protein discovery method based on the integration of protein-protein interaction and gene expression data
Source: BMC Syst Biol. 2012 Mar 10;6:15. doi: 10.1186/1752-0509-6-15 (PMC3325894; doi:10.1186/1752-0509-6-15)
Supplement: Additional file 2 — PeC is compared with fifteen recent centrality measures (DC, DMNC, BC, SC, BN, CC, EC, IC, LAC, SoECC, RL, LI, LR, NC, and MC) by a jackknife methodology. This file includes five figures: (a) PeC is compared with DC and DMNC; (b) PeC is compared with BC, SC and BN; (c) PeC is compared with CC, EC and IC; (d) PeC is compared with LAC and SoECC; (e) PeC is compared with RL, LI, LR, NC, and MC. To compare with the results of random sorting, ten random assortments are also plotted in each figure. The X-axis represents the ranked proteins in the yeast protein-protein interaction network, ranked from left to right as the highest to the lowest values of centrality measures. The Y-axis is the cumulative count of essential proteins with respect to the ranked proteins moving left to right. (DOC 7744 kb). [file 1752-0509-6-15-S2.DOC]

(a) PeC is compared with DC and DMNC

(b) PeC is compared with BC, SC and BN

(c) PeC is compared with CC, EC and IC

(d) PeC is compared with LAC and SoECC

(e) PeC is compared with RL, LI, LR, NC, and MC.

Figure A: PeC is compared with fifteen recent centrality measures (DC, DMNC, BC, SC, BN, CC, EC, IC, LAC, SoECC, RL, LI, LR, NC, and MC) by a jackknife methodology. To compare with the results of random sorting, ten random assortments are also plotted in each figure. The X-axis represents the ranked proteins in the yeast protein-protein interaction network, ranked from left to right as the highest to the lowest values of centrality measures. The Y-axis is the cumulative count of essential proteins with respect to the ranked proteins moving left to right.
