# Supplementary material for: A new essential protein discovery method based on the integration of protein-protein interaction and gene expression data
Source: BMC Syst Biol. 2012 Mar 10;6:15. doi: 10.1186/1752-0509-6-15 (PMC3325894; doi:10.1186/1752-0509-6-15)
Supplement: Additional file 4 — A list of 41 proteins predicted by PeC which are ignored by the ten centrality measures: DC, DMNC, BC, SC, BN, CC, EC, IC, LAC, SoECC when predicting the top 100 proteins. There are some proteins which are ignored by the ten centrality measures: DC, BC, CC, SC, EC, IC, BN, DMNC, LAC, and SoECC, but identified by PeC. This file provides the list of 41 proteins predicted by PeC which are ignored by all the ten centrality measures when predicting the top 100 proteins. (DOC 68 kb). [file 1752-0509-6-15-S4.DOC]

Table B. A list of 41 proteins predicted by PeC which are ignored by the ten centrality measures DC, BC, CC, SC, EC, IC, BN, DMNC, LAC, and SoECC when predicting the top 100 proteins.

| Rank | Proteins | DC | PeC | Essentiality |
| --- | --- | --- | --- | --- |
| 11 | YHR197W | 44 | 11.043 | essential |
| 13 | YCL059C | 34 | 10.422 | essential |
| 14 | YBR247C | 35 | 10.380 | essential |
| 18 | YER082C | 49 | 9.331 | essential |
| 23 | YLR074C | 60 | 8.238 | non-essential |
| 27 | YOL021C | 17 | 7.779 | essential |
| 29 | YOR116C | 27 | 7.674 | essential |
| 30 | YDR060W | 29 | 7.539 | essential |
| 34 | YDR280W | 18 | 7.241 | essential |
| 35 | YJL069C | 35 | 7.219 | essential |
| 37 | YNR003C | 15 | 7.035 | essential |
| 45 | YNL284C | 28 | 6.475 | non-essential |
| 52 | YKL144C | 15 | 5.703 | essential |
| 53 | YDL014W | 33 | 5.620 | essential |
| 58 | YPR103W | 24 | 5.143 | essential |
| 60 | YDR101C | 18 | 4.973 | non-essential |
| 62 | YPL093W | 39 | 4.883 | essential |
| 64 | YHR069C | 12 | 4.846 | essential |
| 66 | YOR361C | 19 | 4.771 | essential |
| 67 | YBR142W | 24 | 4.766 | essential |
| 69 | YNL207W | 28 | 4.692 | essential |
| 70 | YDL111C | 9 | 4.688 | essential |
| 72 | YJR002W | 8 | 4.595 | essential |
| 73 | YJL011C | 6 | 4.583 | essential |
| 77 | YER006W | 23 | 4.503 | essential |
| 78 | YOR206W | 17 | 4.453 | essential |
| 79 | YPR041W | 23 | 4.339 | essential |
| 80 | YHR089C | 22 | 4.337 | essential |
| 81 | YBR079C | 23 | 4.266 | essential |
| 82 | YBL038W | 22 | 4.249 | non-essential |
| 85 | YLR384C | 13 | 4.175 | non-essential |
| 86 | YDL031W | 10 | 4.143 | essential |
| 87 | YDL060W | 18 | 4.139 | essential |
| 92 | YLR129W | 5 | 3.990 | essential |
| 93 | YDR496C | 19 | 3.985 | non-essential |
| 94 | YOR224C | 16 | 3.979 | essential |
| 97 | YOL077C | 10 | 3.957 | essential |
| 98 | YJR065C | 11 | 3.912 | essential |
| 99 | YDR429C | 15 | 3.873 | essential |
| 100 | YPL086C | 7 | 3.858 | non-essential |
