# Supplementary material for: A new essential protein discovery method based on the integration of protein-protein interaction and gene expression data
Source: BMC Syst Biol. 2012 Mar 10;6:15. doi: 10.1186/1752-0509-6-15 (PMC3325894; doi:10.1186/1752-0509-6-15)
Supplement: Additional file 6 — Examples of non-essential proteins which have high degree but with low PeC. Two examples of non-essential proteins YGR254W and YDL059C are shown. YGR254W and YDL059C both have a high degree of 67, but their PeC values are very low. The PeC value of YGR254W is 0.007 and that of YDL059C is -0.241. (DOC 246 kb). [file 1752-0509-6-15-S6.DOC]

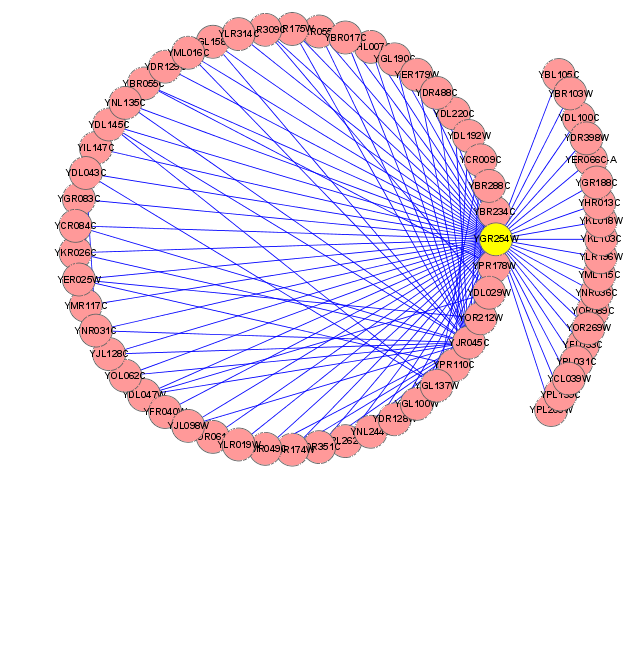

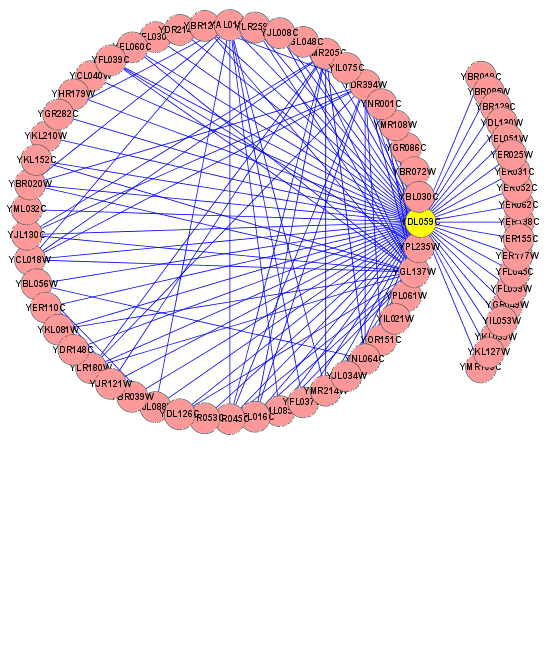


(a) YGR254W (b) YDL059C

Figure B. Examples of non-essential proteins which have high degree but with low PeC. YGR254W and YDL059C both have a high degree of 67, but their PeC values are very low. The PeC value of YGR254W is 0.007 and that of YDL059C is -0.241.
