# Supplementary material for: A new essential protein discovery method based on the integration of protein-protein interaction and gene expression data
Source: BMC Syst Biol. 2012 Mar 10;6:15. doi: 10.1186/1752-0509-6-15 (PMC3325894; doi:10.1186/1752-0509-6-15)
Supplement: Additional file 7 — Examples of non-essential proteins which have high degree and high SoECC but with low PeC. Two examples of non-essential proteins YML048W and YHR140W are shown. YML048W and YHR140W not only have a high degree but also have a high value of SoECC. However, their PeC values are very low. The PeC of YML048W is -0.241 and that of YHR140W is -2.447. (DOC 518 kb). [file 1752-0509-6-15-S7.DOC]

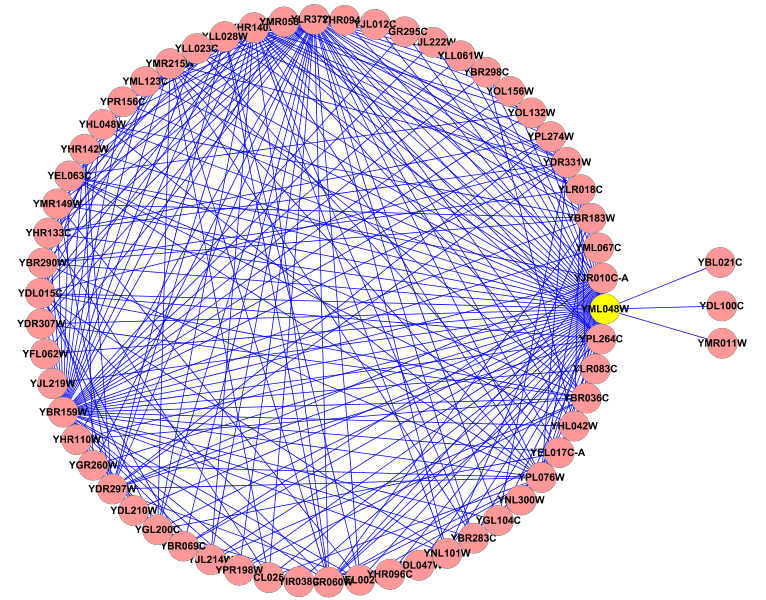

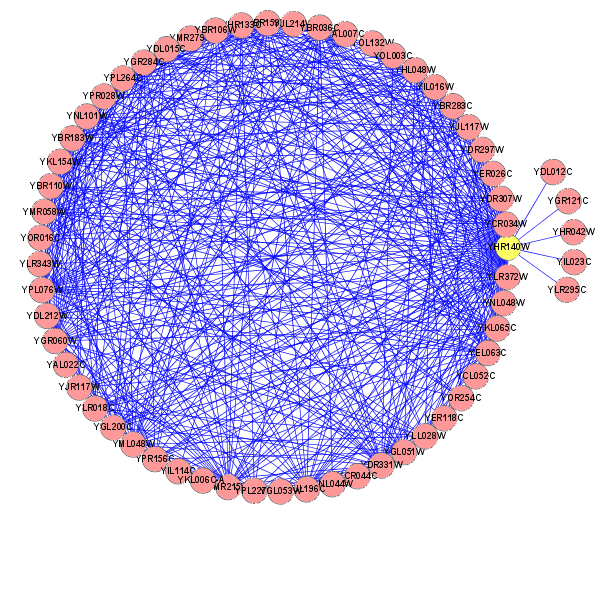


(a) YML048W (b) YHR140W

Figure C. Examples of non-essential proteins which have high degree and high SoECC but with low PeC. YML048W and YHR140W not only have a high degree but also have a high value of SoECC. However, their PeC values are very low. The PeC of YML048W is -0.241 and that of YHR140W is -2.447.
